# Ischemic Stroke Genetics: What Is New and How to Apply It in Clinical Practice?

**DOI:** 10.3390/genes13010048

**Published:** 2021-12-24

**Authors:** Aleksandra Ekkert, Aleksandra Šliachtenko, Julija Grigaitė, Birutė Burnytė, Algirdas Utkus, Dalius Jatužis

**Affiliations:** 1 Center of Neurology, Faculty of Medicine, Vilnius University, LT-03101 Vilnius, Lithuania; julija.grigaite@mf.stud.vu.lt (J.G.); dalius.jatuzis@mf.vu.lt (D.J.); 2 Faculty of Medicine, Vilnius University, LT-03101 Vilnius, Lithuania; aleksandra.sliachtenko@mf.stud.vu.lt; 3 Center for Medical Genetics, Faculty of Medicine, Vilnius University, LT-03101 Vilnius, Lithuania; birute.burnyte@mf.vu.lt (B.B.); algirdas.utkus@mf.vu.lt (A.U.)

**Keywords:** stroke genetics, polygenic stroke risk, genetic stroke causes, monogenic stroke causes, single nucleotide polymorphism

## Abstract

The etiology of ischemic stroke is multifactorial. Although receiving less emphasis, genetic causes make a significant contribution to ischemic stroke genesis, especially in early-onset stroke. Several stroke classification systems based on genetic information corresponding to various stroke phenotypes were proposed. Twin and family history studies, as well as candidate gene approach, are common methods to discover genetic causes of stroke, however, both have their own limitations. Genome-wide association studies and next generation sequencing are more efficient, promising and increasingly used for daily diagnostics. Some monogenic disorders, despite covering only about 7% of stroke etiology, may cause well-known clinical manifestations that include stroke. Polygenic disorders are more frequent, causing about 38% of all ischemic strokes, and their identification is a rapidly developing field of modern stroke genetics. Current advances in human genetics provide opportunity for personalized prevention of stroke and novel treatment possibilities. Genetic risk scores (GRS) and extended polygenic risk scores (PRS) estimate cumulative contribution of known genetic factors to a specific outcome of stroke. Combining those scores with clinical information and risk factor profiles might result in better primary stroke prevention. Some authors encourage the use of stroke gene panels for stroke risk evaluation and further stroke research. Moreover, new biomarkers for stroke genetic causes and novel targets for gene therapy are on the horizon. In this article, we summarize the latest evidence and perspectives of ischemic stroke genetics that could be of interest to the practitioner and useful for day-to-day clinical work.

## 1. Introduction

Stroke is a frequent medical emergency, the burden of which is rising annually [1]. In 2019, there were 12.2 million incident cases of stroke, making it the second-leading death cause in the world and third-leading cause of death and disability combined. Ischemic stroke was the most frequent among incident cases and constituted 62.4% of all strokes [2].

The etiology of ischemic stroke is multifactorial. Traditional modifiable risk factors, such as hypertension, smoking, diabetes and hyperlipidemia, are highlighted frequently [3,4], whereas the role of genetics is usually less accented, though no less substantial. As modern medicine tends to be individualized, personalized prevention and treatment strategies based on a patient’s genetic information have gradually become routine practice [5,6]. It is well known that a genetic component plays an important role in early-onset strokes. Younger onset cases have a stronger genetic burden from common disease-associated single-nucleotide polymorphisms (SNP) [7], thus being the extreme phenotypic expression of the genetic disorder. Less severe or older-onset stroke cases might remain genetically untested due to this bias. In such cases, stroke etiology might be attributed only to traditional modifiable risk factors without further risk stratification according to individual genetic profiles.

To create an appropriate strategy using genetically personalized approach, it is necessary to classify stroke according to genetic risk subtypes. The widely used TOAST classification is based on stroke phenotypical criteria, the main limitation of which is its post factum use; that is, the events are classified after their occurrence. While focusing on using genetic risk for stroke prevention, genetic stroke risk classification is needed. To address this need, several classification systems were proposed [8,9]. Differently from TOAST classification, it is based on genetic information corresponding to various stroke phenotypes, used in TOAST classification (i.e., large-vessel, small-vessel, cardioembolic etc.), which are further categorized into subphenotypes, e.g., extra- and intracranial.

To describe main genetic stroke risk factors, we modified the classification used by Ilinca et al. [9], omitting hemorrhagic stroke risk factors, as our review focuses primarily on the ischemic stroke topic (Table 1). Some factors increase stroke risk directly, while others are linked to stroke risk conditions: hypertension, hyperlipidemia, structural heart abnormalities and hypercoagulative states.

The largest heredity of approximately 40% is seen in large artery stroke (LAS), followed by cardioembolic stroke (CES)—33%. So far only 16% of strokes caused by small-vessel disease (SVD) have been reported to have an etiological genetic component [10,11].

## 2. Methods of Genetic Studies of Stroke

Historically, twin and family history studies, also called linkage analysis, were common methods to determine heritability of stroke [12]. These studies have successfully proved association between stroke and some monogenic disorders (such as CADASIL) [13]. However, they did not prove especially effective in polygenetic research. Twin studies, especially in monozygotic twins, are important in investigating overall influence of genes in the development of a disorder, but they contribute little to a whole study of stroke genetics. On the other hand, family history studies are not that reliable, as their interpretation is obstructed by insufficient data, heterogeneity and possible bias [12]. 

Candidate gene approach is another popular method to discover genetic stroke causes. The essence of this technique is identifying genetic variants in a gene known to be associated with a distinct pathology, a so-called candidate gene [14]. However, this approach has its limitations. The main drawback is its high subjectivity: it is necessary to describe stroke phenotype precisely and then compare it to already available data. However, if the phenotype is not described appropriately or is not characterized by typical featured, the candidate gene might be chosen incorrectly. Moreover, this approach does not help to reveal new genetic variants, as already known stroke-associated genes are being tested. That means no completely new and unknown genetic reasons can be found. What is more, some candidate gene studies do not meet certain standards (like sample size and unbiased publication). Due to this fact and the aforementioned difficulty to identify the phenotype properly not all results are possible to be replicated. [15]

The last method, which is promising, effective and used with increasing frequency, is Genome-Wide Association Studies (GWAS). Genome-wide association studies (GWAS) allow the simultaneous genotyping of more than one million polymorphisms across the genome. In contrast to the candidate gene method, which is also tied to SNP, GWAS studies not a single specific gene, but the entire genome. This allows identifying novel links between chromosomal loci and disease as well as detecting new significant SNPs in already known genes. One potential drawback to GWAS is that these studies are underpowered to detect rare variants. The missing heritability in ischemic stroke subtypes using this method can in part be attributed to low-frequency and rare variants.

Next generation sequencing (NGS) technologies can provide data on complete genomes (whole genome sequencing, WGS), or the coding sequences (whole exome sequencing, WES) [16]. It is mostly used to identify some rare genetic variants which cannot be revealed by GWAS. NGS has now become the preferred technology for most genetic testing in clinics, for example, for cancer diagnosis. [17]. The main challenges and limitations of this technology are relying on clonal PCR and interpretation of a large amount of data. However, relatively low cost and quick application makes NGS a promising tool, not only for research, but also for daily diagnostic of stroke-related inherited disorders [18].

## 3. Known Monogenic Disorders

Some specific genetic alterations follow a Mendelian pattern of inheritance and cause disorders, which can lead to stroke. These are called monogenic disorders, and usually they are related to the specific and rare stroke subtypes. Some of the most frequent monogenic disorders resulting in ischemic stroke or increasing its risk are listed in Table 2 (modified from Falcone et al.) [19].

Despite the fact that monogenic ischemic strokes occur in only 7% of ischemic stroke patients, it is important to know and diagnose the most common syndromes. [20] Cerebral Autosomal Dominant Arteriopathy with Subcortical Infarcts and Leukoencephalopathy (CADASIL) is most common monogenic cause of ischemic stroke [21]. It is caused by a pathogenic variant of *NOTCH3* gene, which is involved in arterial development and is inherited in an autosomal dominant way [22]. Clinical symptoms include young or mid-adult onset of recurrent strokes, migraine with aura, progressing dementia, apathy and psychiatric disorders. Diffuse white matter hyperintensities, external capsule and temporal poles involvement and subcortical infarcts are typical neuroimaging findings [23]. CADASIL is diagnosed either by molecular genetic testing or by skin biopsy, detecting characteristic findings by electron microscope and immunohistochemistry. [24] CARASIL (Cerebral Autosomal Recessive Arteriopathy with Subcortical Infarcts and Leukoencephalopathy) is a clinically similar, but much rarer autosomal recessive disorder. It is caused by *HTRA1* gene mutation, which is thought to maintain cerebral small vessels integrity. Only about 50 cases of CARASIL have been reported, mainly in Japanese males between 20–50 years [25,26]. Beside recurrent strokes, this disease usually manifest in premature head alopecia (about 90%), early vascular dementia and severe back pain with lumbar disk herniation [23].

Fabry’s disease is caused by *GLA* gene mutation resulting in lysosomal enzyme α galactosidase A deficiency [27]. Although originally this X-linked recessive disorder was thought to be phenotypically relevant only in males, it is known now that a heterozygous female carrier might also have similar clinical symptoms [28]. The disorder usually manifests in premature age, causing neuropathies and acroparesthesias. The rest of the clinical spectrum is broad and includes chronic abdominal pain, angiokeratoma, renal dysfunction, cardiac arrhythmias, hypertrophy and other symptoms [29].

Mitochondrial Myopathy, Encephalopathy, Lactic Acidosis, and Stroke-like episodes (MELAS) differs from the aforementioned disorders as it is caused by a variant in mitochondrial DNA (mtDNA) which is inherited maternally [30]. There are several different pathogenic variants, the most common (80%) being m.3243A>G. [31] Phenotypic manifestations are also heterogenic; typical symptoms include seizures, migraine-like headache, ataxia, hearing impairment, diabetes as well as muscle weakness and myopathy. Stroke is reported to occur in 17% of all patients [32].

Retinal vasculopathy with cerebral leukodystrophy (RVCL) is an autosomal dominant disorder caused by mutation in the *TREX1* gene. RVCL affects small vessels and causes progressive vision loss, migraine, psychiatric abnormalities, cognitive impairment, seizures and ischemic strokes. Radiological findings include white matter hyperintensities and subcortical pseudotumors with edema [33].

Forkhead box transcription factor 1 (*FOXC1*) gene plays a role in arterial specification and angiogenesis regulation. It was initially associated with Axenfeld-Rieger Syndrome (ARS), which is presented with ocular, dental and umbilical abnormalities as well as systemic dysmorphism. Multiple case reports have provided a link between *FOXC1* gene and SVD as patients with *FOXC1* deletion have white matter hyperintensities on MRI from 18 months [34].

Other genes related to small-vessel disease resulting in lacunar ischemic and hemorrhagic stroke are *COL4A1* and *COL4A2*; both are located on 13 chromosome (13q34 locus) and encode α1 and α2 chains of type IV collagen [35]. The mutated variants are inherited mainly in autosomal dominant way, although de novo mutation rate is also high (about 40%) [36]. As collagen type IV is an important component of almost all tissues basement membrane, corresponding mutations cause multi-systemic disorders. These include childhood-onset seizures, hemorrhages and porencephaly, adult-onset intracerebral hemorrhages, myopathy, nephropathy, retinopathy and cardiac arrhythmias [37,38]. Similar, but clinically different is the *COL3A1* mutation caused Vascular Ehlers-Danlos syndrome (vEDS) (previously known as type IV). [39] *COL3A1* encodes type III collagen, which is main blood vessels and hollow organs walls protein. Therefore, vEDS typically manifests with fragility of vascular, urinal, intestinal and reproductive systems [40].

Sickle-cell disease is a group of autosomal recessive *HBB* (hemoglobin subunit β) mutation diseases. Pathologic variants of mutant hemoglobin cause hemolysis of erythrocytes, vaso-occlusion and immune system activation, which manifest in acute pain crisis, acute chest syndrome, stroke and some chronic complications [41].

Homocystinuria is caused by cystathionine β-synthase gene mutation, inherited in an autosomal recessive manner, and is characterized by corresponding enzyme deficiency. Clinical manifestation involves retinal, skeletal, vascular and neurological pathology (not necessarily all at once). Sometimes it can even be asymptotic, until an adult-onset cerebral cardioembolic event [42]. 

Marfan syndrome is caused by *FBN1* gene mutation and is characterized by connective tissue disorder [43]. It is best known for cardiovascular and musculoskeletal abnormalities; however other systems are also affected (i.e., ophthalmologic and pulmonary). The most common cause of death in Marfan syndrome is aortic root dissection [44].

Pseudoxanthoma elasticum is a rare autosomal recessive disorder, which is caused by *ABCC6* mutation and consequently ABCC6 protein deficiency [45]. It manifests as mineralization of elastic fibers and their accumulation in the skin, eyes and blood vessels. The most obvious symptom of Pseudoxanthoma elasticum is yellow popular lesions of the skin. However, as mineralization also affects blood vessels, risk of cardiovascular diseases, including strokes, is elevated [46].

## 4. Polygenic Disorders

As claimed by the name, polygenic disorders are caused by multiple genes and do not follow Mendelian inheritance. Each of them has little effect to stroke pathogenesis, but since one person can carry a lot of risk alleles, the overall risk of stroke can be quite high. Compared to the monogenic, polygenic disorders cause about 38% of all ischemic strokes [47]. Identification of polygenic variants is rapidly developing field of stroke genetics. GWAS as well as candidate-gene analyses are the main tools used by researches in search of these genes. A lot of genetic variants were found during GWAS of other cardiovascular diseases.

### 4.1. Cardioembolic Stroke

Although there are multiple genes associated with AF recurrence, not all of them are also associated with stroke. There are not so many genes linking AF and stroke detected so far. Two genes (*PITX2* and *ZFHX3*), located in chromosomes 4q25 and 16q22 were identified to be significant risk factors of AF and CE stroke [48,49]. SNPs in *PITX2* and *ZFHX3* genes increase risk of CE stroke by 36% and 25%, respectively. The significance of those genes was repeatedly reconfirmed by other studies, adding two other genes significantly associated with CES to the stroke genes archive: *ZNF566* and *PDZK1IP1* [50,51]. 

### 4.2. Large Artery Stroke

With regards to large artery stroke (LAS) subtype, there are also some relevant findings. The main variant within the 7p21 chromosome, which is close to the *HDAC9* gene, is associated with a 39% increase of LAS strokes [8,52]. The exact mechanism of stroke risk is not yet clear, but there are few hypotheses considering atherosclerosis acceleration and altering brain ischemic responses [53]. Another gene associated with LAS is *CDKN2A/CDKN2B* [53]. It is situated at 9p21 and increases the risk of ischemic stroke by 15% [19]. One more important gene for LAS is *MMP12*, which encodes matrix metalloproteinases and is a part of MMP genes cluster, localized in chromosome 11. Interestingly, this gene is overexpressed in the carotid plaques [54]. The NINDS SiGN (National Institute of Neurological Disorders and Stroke Genetics Network) investigators detected a SNP at 1p13.2 near *TSPAN2* that was associated with increased LAS risk, whereas the *TSPAN2* gene itself is associated with migraine and retinal vasculopathy with cerebral leukoencephalopathy and systemic manifestations. *TSPAN2* is expressed in arterial tissue and in blood cells, and *Tspan2*-deficient mice demonstrate increased neuroinflammation with activation of microglia and astrocytes. [8,52].

A large study from MEGASTROKE consortium has shown that genetic predisposition to higher concentration of low-density lipoprotein (LDL) particles and overall higher LDL-cholesterol concentrations were associated with higher risk of large artery stroke, while the risk of genetically determined higher ApoB concentration was not confirmed [55]. A significant degree of shared polygenic risk between lung function and ischemic stroke (IS) was another interesting finding of the study, being explained with systemic inflammation and oxidative stress in chronic obstructive pulmonary disease, promoting cerebral vascular dysfunction and platelet hyperactivity.

### 4.3. Small Vessel Disease

There are two subtypes of lacunar stroke, both of which can be precipitated by genetic risk factors: isolated lacunar stroke and multiple lacunar stroke with leucoaraiosis. Multiple studies revealed different genetic mechanisms increasing risk for lacunar strokes: impaired oxidative phosphorylation pathways and various single nucleotide polymorphisms, some of them located in genes implicated in Alzheimer’s disease and intracerebral hemorrhage [52].

The CHARGE (Cohorts for Heart and Aging Research in Genomic Epidemiology) Consortium reported on a locus on chromosome 6p25 near the *FOXF2* gene that reached genome-wide significance for all stroke subtypes and white matter hyperintensity (WMH) burden [56]. Single nucleotide polymorphism within 16q24.2 locus has also been found to be associated with small-vessel stroke. The same locus also influences WMH, however, it does not appear to increase risk of intracerebral hemorrhage. Associations of the locus with expression of *ZCCHC14* and DNA methylation suggest the locus acts through changes to regulatory elements [7]. The *SH2B3* gene at a 12q24 locus, originally associated with all ischemic stroke but not with any specific subtype, exceeded genome-wide significance in the meta-analysis of small artery stroke [8].

White matter hyperintensity (WMH) volume is a commonly used marker to identify small vessel disease and measure its burden. Some authors choose it as primary endpoint instead of lacunar stroke occurrence. A study examining WMH volume, instead of LS specifically, found several associated SNPs involving the genes *TRIM65* and *TRIM47* [57]. 

As it was already mentioned, genetic studies are extremely important while dealing with early-onset stroke. A UMD-GEOS Study comparing subjects who had stroke at the age 15–49 years with non-stroke controls found significant *NAT10* gene burden in early-onset small vessel stroke. As this gene also plays an important role in progeria and laminopathies, the authors claim that it would be reasonable to study its role in early-onset stroke further. [58]. Sargurupremraj et al. conducted a large multi-ancestry meta-analysis of WMH genome-wide association studies (GWAS), accounting for hypertension as a potential confounder and effect modifier [59]. They analyzed more than 50,000 older subjects and discovered 27 novel loci responsive for white matter hyperintensities. Further analysis of younger adults in their twenties has shown that the same loci were associated with early changes in white matter microstructure on MRI using diffusion tensor imaging (DTI). Higher levels of blood pressure (BP) were likely causally associated with larger WMH volume even while below the hypertension threshold, currently defined as 140/90 mmHg.

Other genes, linked to SVD, are *CSN3* and *HLA-DPB1*, which are unique to patients with lacunar stroke, *CSN3*, which is also associated with coronary disease and diabetes mellitus, and *SH3TC1*, which is also implicated in Charcot-Marie-Tooth disease [57]. 

### 4.4. Other and Multiple Stroke Types

EuroCLOT study have detected that ABO gene, which is associated with circulating levels of von Willebrand factor and factor VIII, was confined to large artery stroke and cardioembolic stroke, but not small-vessel stroke [60]. ABO gene SNPs increase risk of CES and LAS by 13% and 12%, respectively [19,60]. 

Data from Swedish cohort highlighted the importance of factor VII activating protease (FSAP) for stroke risk. The authors reconfirmed the significance of HABP2 gene and found a novel significant ADCY2 gene at a 5p15 locus [61]. The factor VII activating protease (FSAP) knockout mice have a bigger neointima after vascular injury and a larger infarct volume after stroke [62].

Variants associated with all type of strokes were found near FOXF2 (6p25) and ALDH2 (12q24) genes [56,63]. There is also a locus at 12p13 chromosome near NINJ2 gene, which also has possible relation to multiple stroke subtypes [64]. A large multi-ancestry study from MEGASTROKE consortium revealed 32 loci associated with genetic stroke risk, 22 of which were novel, and some new insights about already known genes were obtained. While the ZNF318 variant was thought to increase only the risk of LAS, findings from MEGASTROKE Consortium revealed its association to all types of strokes [65]. The summary of genes specifically increasing the risk for ischemic stroke is provided in Table A1 (Appendix A).

Some genes (CELSR1, PRKCH, PTCSC3, C1ORF156, XYLB) are associated with ischemic stroke specifically in Asian populations [57,66,67,68]. 1425G/A polymorphism in PRKCH has the strongest link to lacunar stroke [69]. Although the results of the study from MEGASTROKE Consortium demonstrated significant correlations of risk-allele frequencies and odds ratios between European and East Asian populations, six loci exhibited population-specific association.

Of course, there are many other genes, corresponding to the ischemic stroke, such as inflammatory (i.e., HLA and killer cell immunoglobulin-like receptor alleles) [70,71]. Some other factors like diet seem to modulate cerebrovascular risk factors on epigenetic level, affecting incidence of ischemic stroke [72]. However, these topics are out of the scope of this article. 

## 5. Discussion

Genetic studies on strokes provide us with new significant information that could potentially contribute to personalized stroke care. Although these findings might seem hardly applicable in clinical practice at the first glance, major breakthrough is evident as genetic risk scores and gene therapies are developing. Numerous genetic risk scores have been already proposed for various conditions, including stroke [73]. Genetic risk scores (GRS) that estimate a cumulative contribution of genetic factors to a specific outcome can be extended to polygenic risk scores (PRS) by taking into account all known genetic markers possibly correlated to the outcome, covering even the loci of the small effects that do not reach genome-wide significance. In 2014, Malik et al. created a multilocus GRS for stroke. They found that combining genetic risk score data with Framingham risk score was significantly associated with ischemic stroke risk. However, its power for predicting a stroke was still limited and did not differ substantially from the power of traditional scores based on clinical risk factors [74]. Similar findings were reported by Swedish authors, who conducted three studies related to hypertension genetic risk factors and ischemic stroke risk. GRS for hypertension was significantly associated with the stroke risk, although it did not perform better while predicting the stroke compared to the clinical risk factor of hypertension itself [75]. Another study conducted in an Asian population showed that the PRS created by the authors predicted a significant stroke risk independently of environmental risk factors [76]. What is the reason for relatively minor predictive value of GRS? It is worth noting that, so far, the studies were concentrated on the risk of overall stroke, while the risk of specific stroke subtypes was not analysed. A study conducted in 2021 evaluated the risk of stroke in subjects with cardiometabolic disease compared to CHA_2_DS_2_-VASc score. GRS containing 32 SNPs was a strong, independent predictor of ischemic stroke. In patients with atrial fibrillation but lower CHA_2_DS_2_-VASc scores, the GRS identified patients with risk comparable to those with higher CHA_2_DS_2_-VASc scores [77]. As can be seen, a more specific population was chosen in this study. It is also interesting that although there are lots of genes linked to increased AF risk, not all of them are associated with the ischemic stroke risk. This might be due to the fact that those gene variants are too rare to detect their impact for stroke risk so far. Nevertheless, if we found in future that some of those genes are linked to AF, but not to CES, it could be a major gamechanger in the field of cardioembolic stroke prevention. Moreover, as novel stroke risk loci are being detected and more information is gained on their individual impact and their interconnections, the precision of GRS and PRS increases.

Despite the fact that genetic risk scores might be less useful when patient already has clinical risk factors, they could provide us with useful insights for primary stroke prevention. In young people with genetic risk factors, earlier and more intensive prevention and treatment strategies could be applied before the clinical risk factors become evident and cause deleterious consequences.

Ilinca et al. have created stroke gene panels for research and clinical practice. The clinical panel includes 61 genes related to stroke directly and 27 more genes related to disorders causing stroke that might be relevant to consider their evaluation in clinical practice. The authors encourage the use of their panels for stroke risk evaluation and further stroke research [9]. 

Another benefit of detecting stroke risk genes is that they could be potential targets for gene therapy in future. HDAC inhibitors have been postulated as a treatment for stroke [53]. One study in knock-out mice suggests a new strategy for acute stroke treatment by suppressing HDAC2 in peri-infarct zone. The authors claim that application of HDAC inhibitors from five to seven days after stroke enhances cell survival and neuroplasticity as well as reduces inflammation, which could potentially provide a wider therapeutic window for stroke recovery [78]. Systemic administration of an agonist NOTCH3 antibody was studied in transgenic mice and showed protective effects against impaired cerebral blood flow [79]. Other genetic stroke risk studies implicated messengerRNA (mi-RNA) as a potential drug target. Zou et al. detected five miRNAs to be potential biomarkers or therapeutic targets for CES, particularly miR-27a-3p, miR27b-3p, and miR-494-3p [50]. Transcriptome-wide colocalization analyses showed association of WMH-volume with expression of 39 genes, of which four encode known drug targets [59].

## 6. Conclusions

Current advances in human genetics combined with relatively modest costs allow clinicians to prevent stroke earlier and classify different stroke subtypes according to their etiology prior to the occurrence of clinical risk factors. This is especially important for young patients who have a genetic predisposition for stroke without clinical manifestations. More precise strategies of constructing GRS for IS and combining GRS with risk factor profiles and clinical information might eventually result in better risk prediction. Detection of novel therapeutic targets and development of corresponding monoclonal antibodies might be a revolution in personalized stroke care.

## Figures and Tables

**Table 1:** Ischemic stroke subtypes classification by genetic risk, modified from Ilinca et al.

Large Artery Atherosclerosis	Unspecified Hypercholesterolemia Hypertension
Large artery structural abnormalities	Tortuosity/dolichoectasia Dissection Occlusion: Moyamoya-like/ fibromuscular dysplasia
Small-vessel disease	Isolated lacunar infarct Multiple lacunar infarcts White matter hyperintensities Hypertension
Cardioembolic	Arrhythmia: atrial fibrillation/flutter Morphological defect, such as patent foramen ovale Myopathy
Coagulopathy	Venous thrombosis Arterial thrombosis Hyperviscosity
Metabolic	Mitochondrial Defect of intermediary metabolism

**Table 2:** Monogenic disorders that include stroke in their phenotypic manifestation (modified from Falcone et al.).

Disorder	Gene	Inheritance	Stroke Mechanism	Clinical Manifestation	Diagnostic Test
CADASIL	*NOTCH3*	Autosomal dominant	SVD	Migraine with aura, recurrent strokes	Molecular genetic tests, skin biopsy
CARASIL	*HTRA1*	Autosomal recessive	SVD	Recurrent strokes, vascular dementia, severe back pain, premature alopecia	Molecular genetic tests
Fabry’s disease	*GAL*	X-linked	Large-artery disease, SVD	Neuropathic, abdominal pain, angiokeratoma, renal and cardiac failure,	Molecular genetic tests, α galactosidase activity
MELAS	mtDNA	Maternal	Complex (microvascular and neuronal factors)	Seizures, headache, ataxia, hearing loss, muscle weakness	Muscle biopsy, mutational analysis of mtDNA
RVCL	*TREX1*	Autosomal dominant	SVD	Visual loss, migraines, cognitive impairment, strokes	Molecular genetic tests
FOXC1-deletion related SVD	*FOXC1*	De novo or inherited mutations, reciprocal translocations	SVD	Subcortical infarcts, ARS, hearing impairment, cerebellar malformations	Molecular genetic tests
COL4A1-A2 syndromes	*COL4A1* and *COL4A2*	Autosomal dominant	SVD	Lacunar infarcts, hemorrhages, including cerebral, developmental delay, seizures, migraine without aura, visual loss, nephropathy, myopathy arrhythmias	Molecular genetic tests
vEDS	*COL3A1*	Autosomal dominant	Arterial dissection	Easy bruising, thin skin with visible veins, characteristic facial features, arterial, uterine or intestinal ruptures	Biochemical analysis, molecular genetic tests
Sickle-cell disease	*H88*	Autosomal recessive	Large-artery disease, SVD, hemodynamic insufficiency	Pain crises, seizures, myelopathy, anemia, bacterial infection, pulmonary, abdominal and vaso-occlusive crises	Peripheral blood smear, electrophoresis, mutational analysis
Homocystinuria	*CBS*	Autosomal recessive	Large-artery disease, CE, SVD, arterial dissection	Mental retardation, atraumatic dislocation of lenses, Marfan-like skeletal deformations, premature atherosclerosis, thromboembolic	Urine analysis, homocysteine and methionine in plasma measurementsMolecular genetic tests
Marfan syndrome	*FBN1*	Autosomal dominant	CE and arterial dissection	Pectus carinatum or excavatum, upper-to-lower segment ratio <0.86, or arm-span-to-height ratio >1.5; scoliosis >20%; ectopia lentis; dilation or dissection of the ascending aorta; lumbosacral dural ectasia	Clinical diagnosisMolecular genetic tests
Pseudoxanthoma elasticum	*ABCC6*	Autosomal recessive	Large-artery disease and SVD	Increased elasticity and yellow-orange popular lesions of skin, ocular changes (angioid streaks), hypertension	Skin biopsy, molecular genetic tests
Disorder	Gene	Inheritance	Stroke mechanism	Clinical manifestation	Diagnostic test
CADASIL	*NOTCH3*	Autosomal dominant	SVD	Migraine with aura, recurrent strokes	Molecular genetic tests, skin biopsy
CARASIL	*HTRA1*	Autosomal recessive	SVD	Recurrent strokes, vascular dementia, severe back pain, premature alopecia	Molecular genetic tests
Fabry’s disease	*GAL*	X-linked	Large-artery disease, SVD	Neuropathic, abdominal pain, angiokeratoma, renal and cardiac failure,	Molecular genetic tests, α galactosidase activity
MELAS	mtDNA	Maternal	Complex (microvascular and neuronal factors)	Seizures, headache, ataxia, hearing loss, muscle weakness	Muscle biopsy, mutational analysis of mtDNA
RVCL	*TREX1*	Autosomal dominant	SVD	Visual loss, migraines, cognitive impairment, strokes	Molecular genetic tests
FOXC1-deletion related SVD	*FOXC1*	De novo or inherited mutations, reciprocal translocations	SVD	Subcortical infarcts, ARS, hearing impairment, cerebellar malformations	Molecular genetic tests
COL4A1-A2 syndromes	*COL4A1* and *COL4A2*	Autosomal dominant, de novo mutation	SVD	Hemorrhages, including cerebral, hemiparesis, developmental delay, seizures, lacunar infarcts, migraine without aura, visual loss, nephropathy, myopathy arrhythmias	Molecular genetic tests
vEDS	*COL3A1*	Autosomal dominant	Arterial dissection	Easy bruising, thin skin with visible veins, characteristic facial features, arterial, uterine or intestinal ruptures	Biochemical analysis, molecular genetic tests
Sickle-cell disease	*H88*	Autosomal recessive	Large-artery disease, SVD, hemodynamic insufficiency	Pain crises, seizures, myelopathy, anemia, bacterial infection, pulmonary, abdominal and vaso-occlusive crises	Peripheral blood smear, electrophoresis, mutational analysis
Homocystinuria	*CBS*	Autosomal recessive	Large-artery disease, CE, SVD, arterial dissection	Mental retardation, atraumatic dislocation of lenses, Marfan-like skeletal deformations, premature atherosclerosis, thromboembolic	Urine analysis, homocysteine and methionine in plasma measurements(molecular genetic tests)
Marfan’s syndrome	*FBN1*	Autosomal dominant	CE and arterial dissection	Pectus carinatum or excavatum, upper-to-lower segment ratio <0.86, or arm-span-to-height ratio >1.5; scoliosis >20%; ectopia lentis; dilation or dissection of the ascending aorta; lumbosacral dural ectasia	Clinical diagnosis(molecular genetic tests)
Pseudoxanthoma elasticum	*ABCC6*	Autosomal recessive	Large-artery disease and SVD	Increased elasticity and yellow-orange popular lesions of skin, ocular changes (angioid streaks), hypertension	Skin biopsy, molecular genetic tests

CADASIL—cerebral autosomal dominant arteriopathy with subcortical infarcts and leukoencephalopathy. CARASIL—cerebral autosomal recessive arteriopathy with subcortical infarcts and leukoencephalopathy. MELAS—mitochondrial myopathy, encephalopathy, lactic acidosis, and stroke-like episodes. mtDNA—mitochondrial DNA; RVCL—retinal vasculopathy with cerebral leukodystrophy; vEDS—Vascular Ehlers-Danlos syndrome; SVD—small vessel disease; CE—cardioembolic.

## Data Availability

Not applicable.

## Appendix A

**Table A1:** Genes contributing to polygenic risk of the ischemic stroke.

Stroke Subtype	Candidate Gene	Chromosome	Polymorphisms	Reference
Large artery stroke	*HDAC9*	7p21.1	rs2107595; rs11984041	PMID: 26708676
	*CDKN2A/CDKN2B*	9p21.3	rs23832073	PMID: 22306652
	*EDNRA*	4q31	rs17612742	PMID: 30356112
	*TM4SF4-TM4Sn*	3q25	rs7610618	PMID: 32874429
	*LINC01492*	9q31	rs10990643	PMID: 31306060
Cardioembolic stroke	*PITX2*	4q25	rs2200733; rs10033464; rs2723334	PMID: 18991354 PMID: 26935894
	*ZFHX3*	16q22.3	rs7193343-T; rs12932445	PMID: 19597491 PMID: 26935894
	*ZNF566*	19q13.12	N/A	
	*PDZK1IP1*	1p33	N/A	
	*RGS7*	1q43	rs146390073	PMID: 32874429
	*NKX2-5*	5q35	rs6891174	PMID: 32874429
Other and multiple types of strokes	*HABP2*	10q25.3	rs41292628	PMID: 30070759
	*ADCY2*	5p15	rs35510613	PMID: 30070759
	*ANK2*	4q25	rs34311906	PMID: 32874429
	*FGA*	4q31	rs6825454	PMID: 32874429
	*LOC100505841*	5q23	rs2303655	PMID: 33293549
	*CDK6*	7q21	rs42039	PMID: 32874429
	*PDE3A*	12p12	rs7304841	PMID: 32874429
	*FURIN-FES*	15q26	rs4932370	PMID: 32874429
	*PRPF8*	17p13	rs11867415	PMID: 32874429
	*ILF3-SLC44A2*	19p13	rs2229383	PMID: 32874429
	*ABO*	9q34	rs505922	PMID: 23381943
	*MMP12*	11q22	rs660599	PMID: 25078452
	*SH2B3*	12q24	rs3184504	PMID: 32874429
All types of strokes (ischemic and hemorrhagic)	*CASZ1*	1p36	rs880315	PMID: 32874429
	*WNT2B*	1p13	rs12037987	PMID: 32874429
	*KCNK3*	2p23	rs12476527	PMID: 32874429
	*SLC22A7-ZNF318*	6p21	rs16896398	PMID: 29531354
	chr9p21	9p21	rs7859727	PMID: 32874429
	*SH3PXD2A*	10q24	rs4630220	PMID: 33293549
	*TBX3*	12q24	rs35436	PMID: 32874429
	*LRCH1*	13q14	rs9526212	PMID: 32874429
	*SMARCA4-LDLR*	19p13	rs8103309	PMID: 32874429
	*PMF1*-*SEMA4A*	1q22	rs1052053	PMID: 32874429
	*FOXF2*	6p25	rs12204590	PMID: 27068588
	*ZCCHC14*	16q24.2	rs12445022	PMID: 27997041
